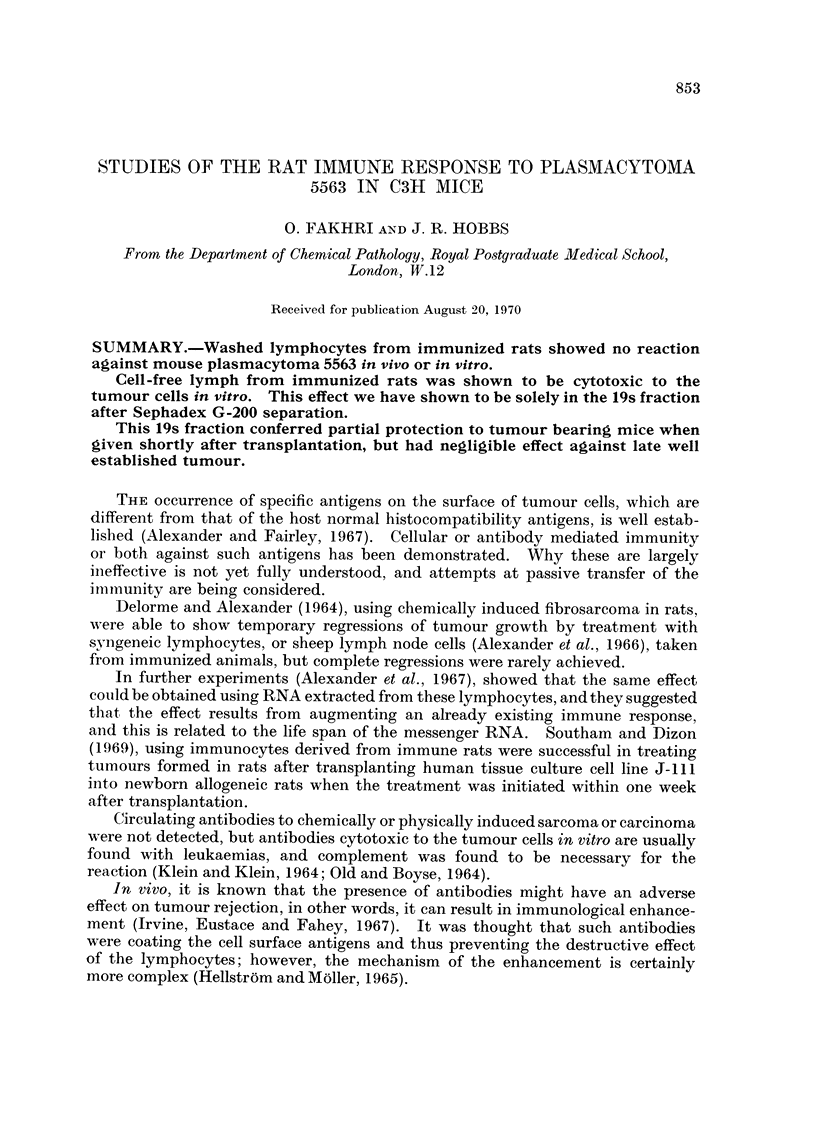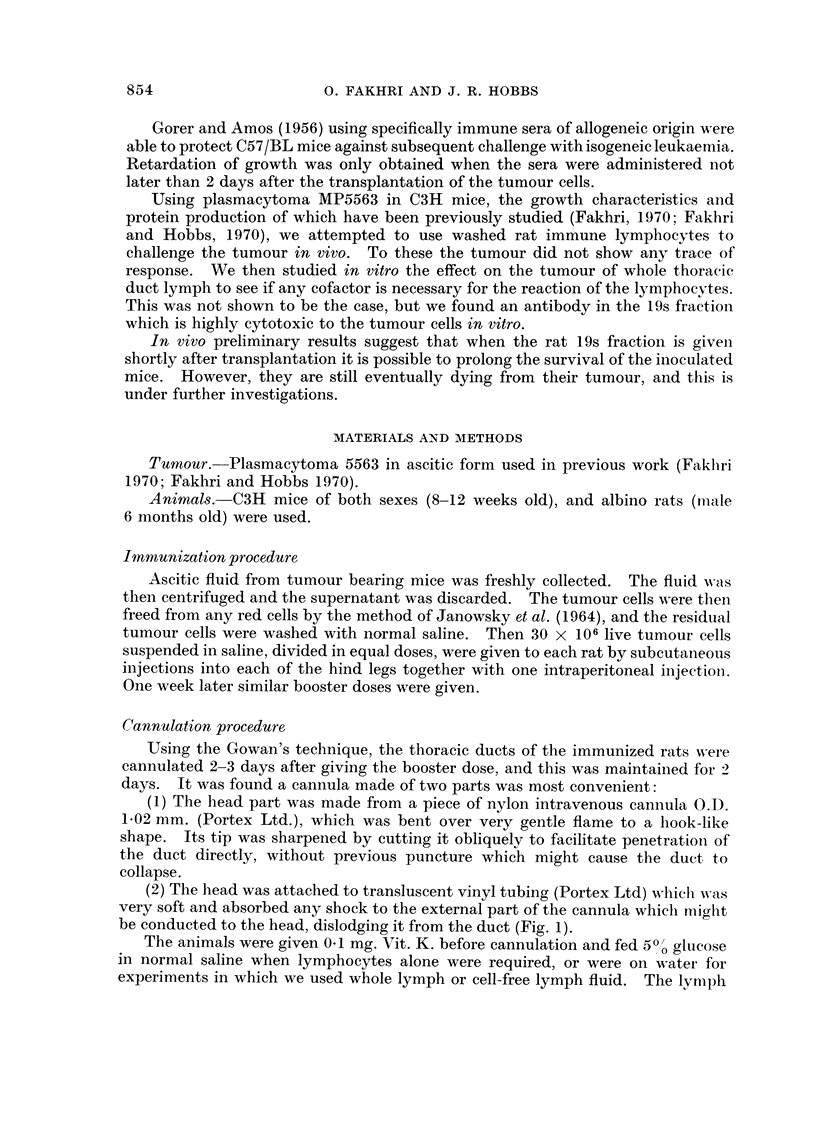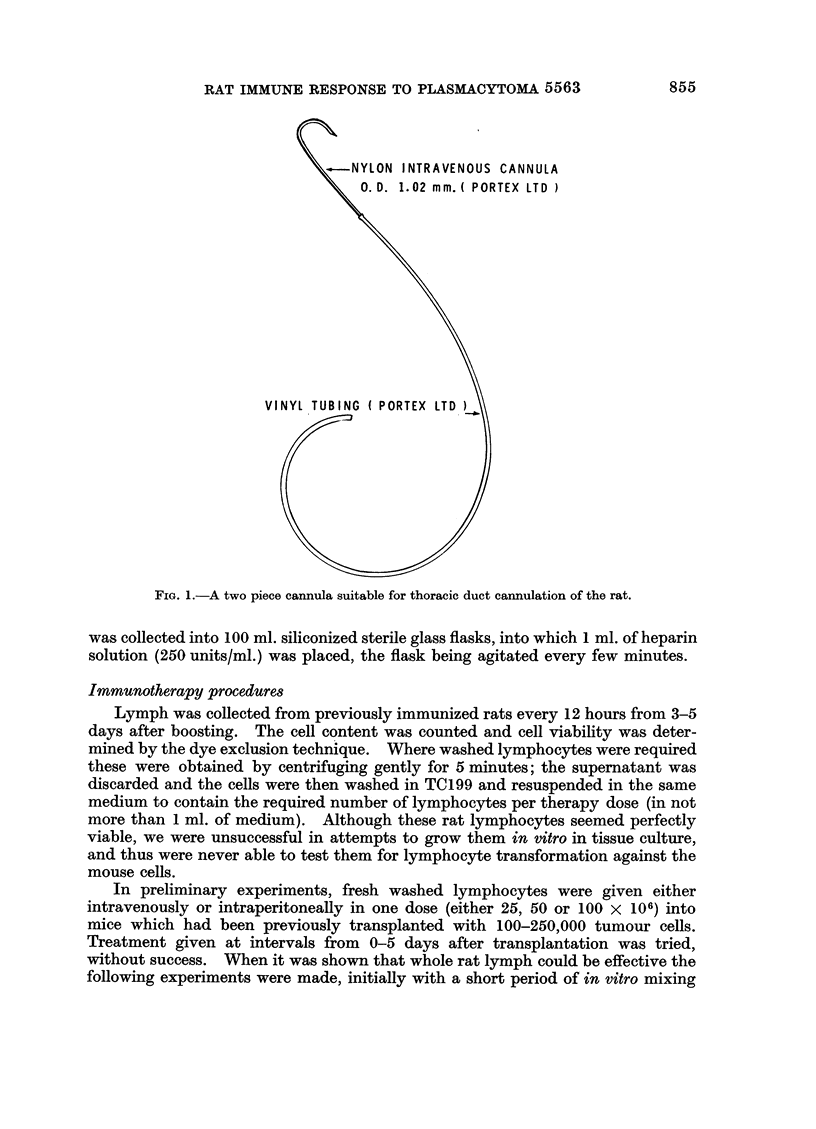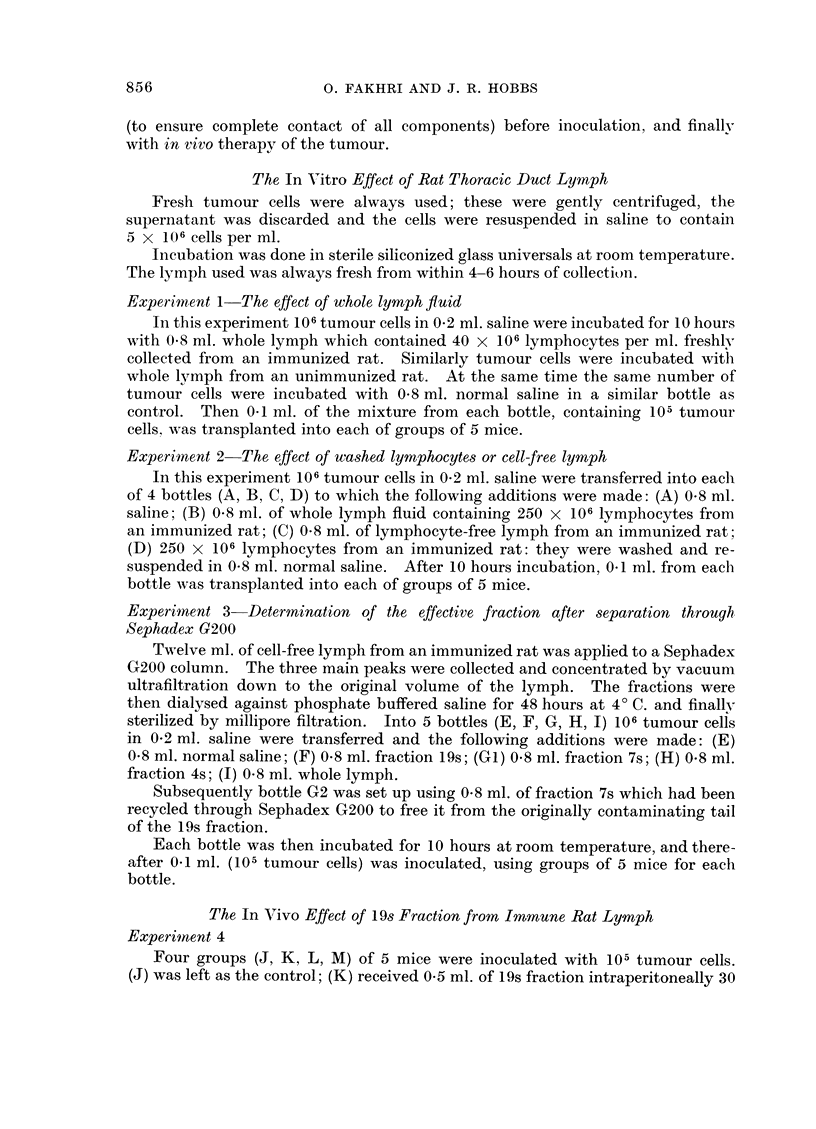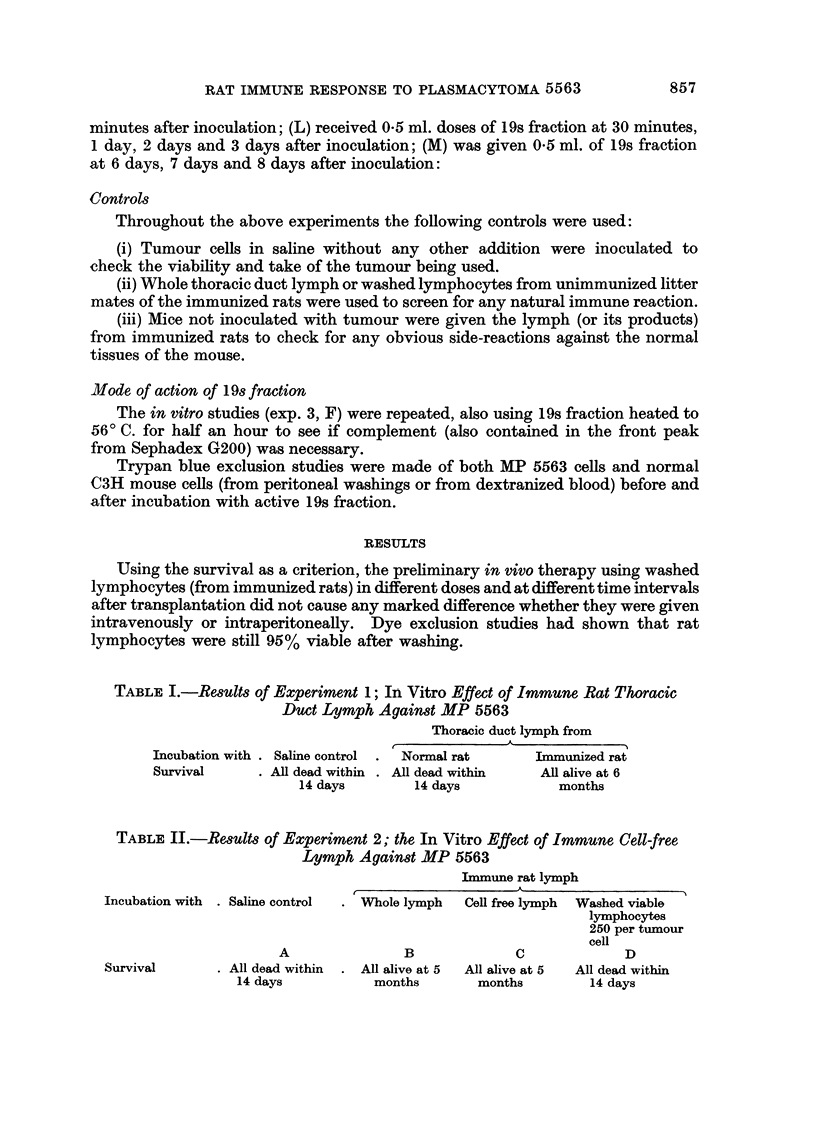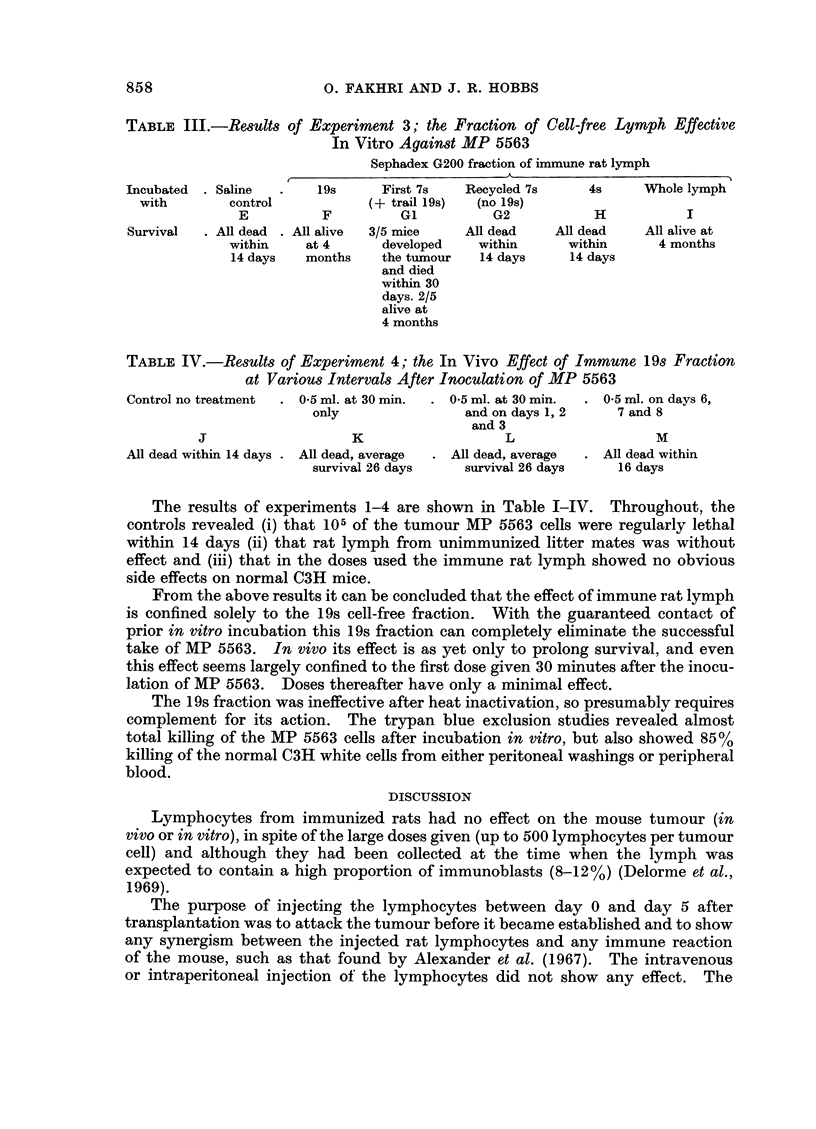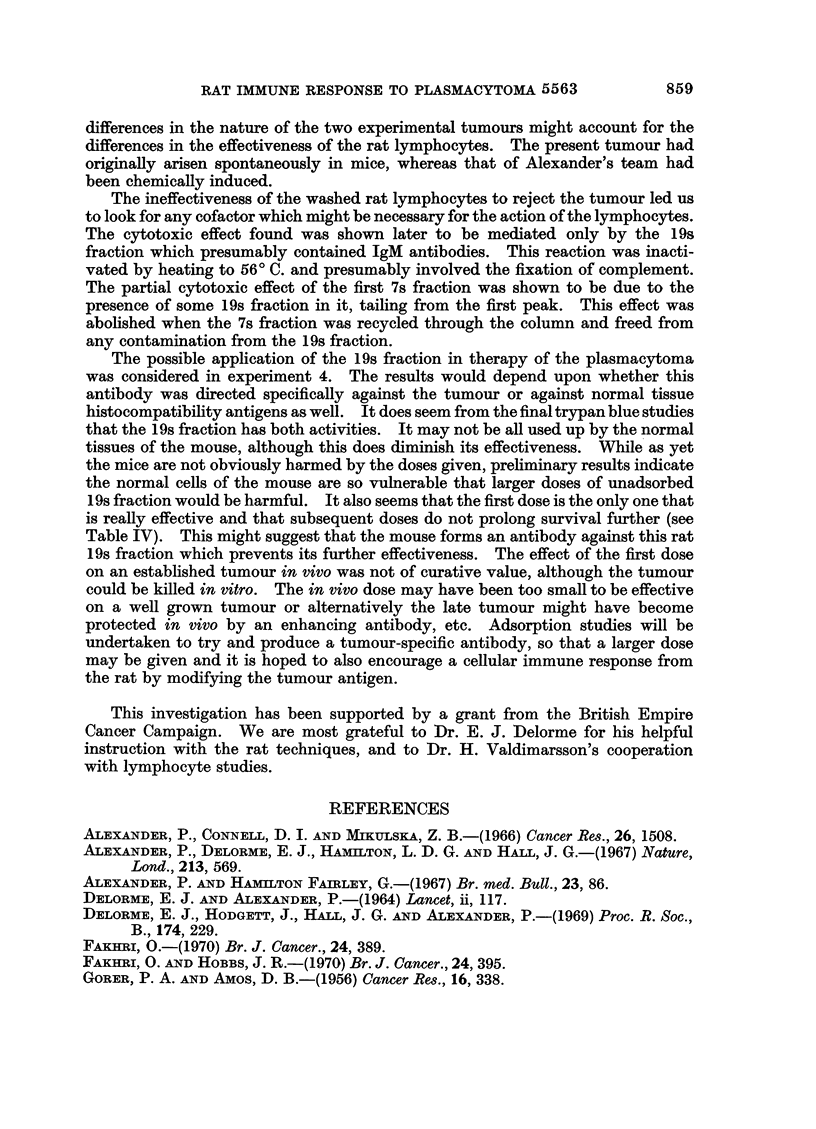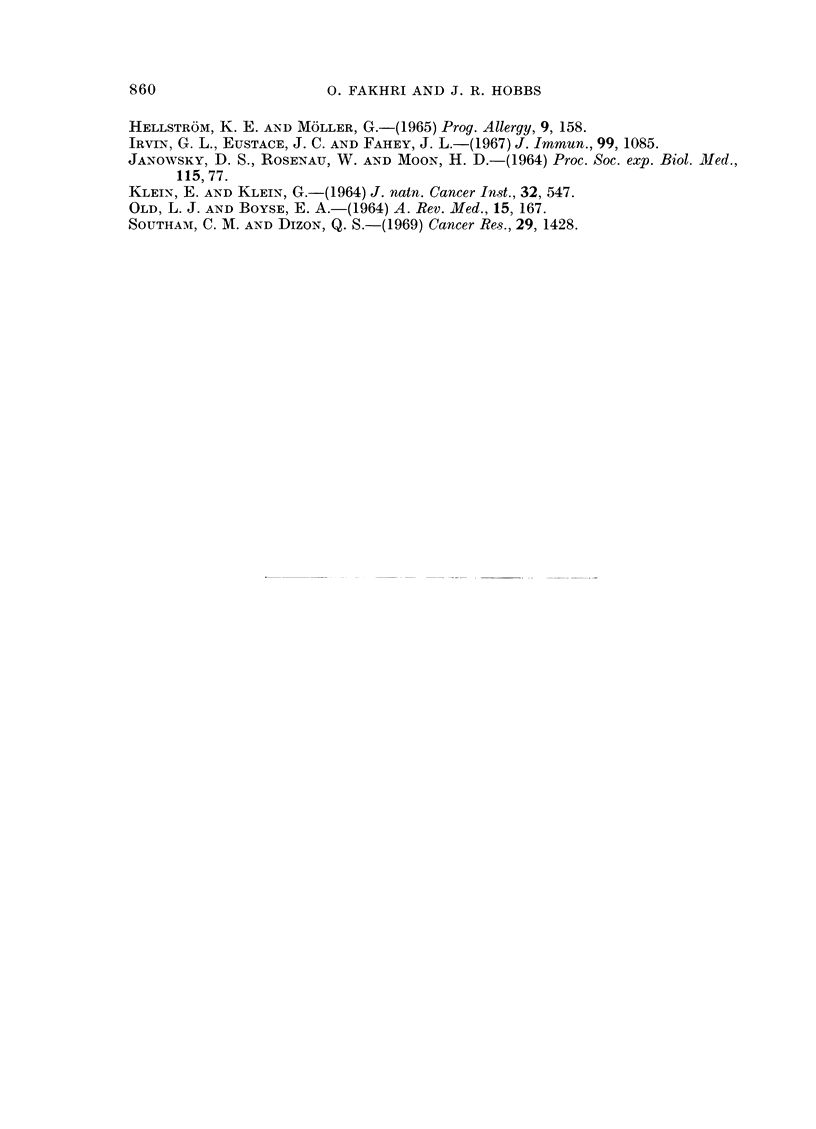# Studies of the Rat Immune Response to Plasmacytoma 5563 in C3H Mice

**DOI:** 10.1038/bjc.1970.101

**Published:** 1970-12

**Authors:** O. Fakhri, J. R. Hobbs

## Abstract

Washed lymphocytes from immunized rats showed no reaction against mouse plasmacytoma 5563 *in vivo* or *in vitro*.

Cell-free lymph from immunized rats was shown to be cytotoxic to the tumour cells *in vitro*. This effect we have shown to be solely in the 19s fraction after Sephadex G-200 separation.

This 19s fraction conferred partial protection to tumour bearing mice when given shortly after transplantation, but had negligible effect against late well established tumour.


### STUDIES OF THE RAT IMMUNE RESPONSE TO PLASMACYTOMA 5563 IN C3H MICE

O. FAKHRI AND J. R. HOBBS

*From the Department of Chemical Pathology, Royal Postgraduate Medical School, London, W.12*

Received for publication August 20, 1970

**SUMMARY.—Washed lymphocytes from immunized rats showed no reaction against mouse plasmacytoma 5563 *in vivo* or *in vitro*.**

**Cell-free lymph from immunized rats was shown to be cytotoxic to the tumour cells *in vitro*. This effect we have shown to be solely in the 19s fraction after Sephadex G-200 separation.**

**This 19s fraction conferred partial protection to tumour bearing mice when given shortly after transplantation, but had negligible effect against late well established tumour.**

THE occurrence of specific antigens on the surface of tumour cells, which are different from that of the host normal histocompatibility antigens, is well established (Alexander and Fairley, 1967). Cellular or antibody mediated immunity or both against such antigens has been demonstrated. Why these are largely ineffective is not yet fully understood, and attempts at passive transfer of the immunity are being considered.

Delorme and Alexander (1964), using chemically induced fibrosarcoma in rats, were able to show temporary regressions of tumour growth by treatment with syngeneic lymphocytes, or sheep lymph node cells (Alexander *et al.*, 1966), taken from immunized animals, but complete regressions were rarely achieved.

In further experiments (Alexander *et al.*, 1967), showed that the same effect could be obtained using RNA extracted from these lymphocytes, and they suggested that the effect results from augmenting an already existing immune response, and this is related to the life span of the messenger RNA. Southam and Dizon (1969), using immunocytes derived from immune rats were successful in treating tumours formed in rats after transplanting human tissue culture cell line J-111 into newborn allogeneic rats when the treatment was initiated within one week after transplantation.

Circulating antibodies to chemically or physically induced sarcoma or carcinoma were not detected, but antibodies cytotoxic to the tumour cells *in vitro* are usually found with leukaemias, and complement was found to be necessary for the reaction (Klein and Klein, 1964; Old and Boyse, 1964).

*In vivo*, it is known that the presence of antibodies might have an adverse effect on tumour rejection, in other words, it can result in immunological enhancement (Irvine, Eustace and Fahey, 1967). It was thought that such antibodies were coating the cell surface antigens and thus preventing the destructive effect of the lymphocytes; however, the mechanism of the enhancement is certainly more complex (Hellström and Möller, 1965).

Gorer and Amos (1956) using specifically immune sera of allogeneic origin were able to protect C57/BL mice against subsequent challenge with isogeneic leukaemia. Retardation of growth was only obtained when the sera were administered not later than 2 days after the transplantation of the tumour cells.

Using plasmacytoma MP5563 in C3H mice, the growth characteristics and protein production of which have been previously studied (Fakhri, 1970; Fakhri and Hobbs, 1970), we attempted to use washed rat immune lymphocytes to challenge the tumour *in vivo*. To these the tumour did not show any trace of response. We then studied *in vitro* the effect on the tumour of whole thoracic duct lymph to see if any cofactor is necessary for the reaction of the lymphocytes. This was not shown to be the case, but we found an antibody in the 19s fraction which is highly cytotoxic to the tumour cells *in vitro*.

*In vivo* preliminary results suggest that when the rat 19s fraction is given shortly after transplantation it is possible to prolong the survival of the inoculated mice. However, they are still eventually dying from their tumour, and this is under further investigations.

### MATERIALS AND METHODS

*Tumour.*—Plasmacytoma 5563 in ascitic form used in previous work (Fakhri 1970; Fakhri and Hobbs 1970).

*Animals.*—C3H mice of both sexes (8–12 weeks old), and albino rats (male 6 months old) were used.

### *Immunization procedure*

Ascitic fluid from tumour bearing mice was freshly collected. The fluid was then centrifuged and the supernatant was discarded. The tumour cells were then freed from any red cells by the method of Janowsky *et al.* (1964), and the residual tumour cells were washed with normal saline. Then $30 \times 10^6$ live tumour cells suspended in saline, divided in equal doses, were given to each rat by subcutaneous injections into each of the hind legs together with one intraperitoneal injection. One week later similar booster doses were given.

### *Cannulation procedure*

Using the Gowan's technique, the thoracic ducts of the immunized rats were cannulated 2–3 days after giving the booster dose, and this was maintained for 2 days. It was found a cannula made of two parts was most convenient:

(1) The head part was made from a piece of nylon intravenous cannula O.D. 1·02 mm. (Portex Ltd.), which was bent over very gentle flame to a hook-like shape. Its tip was sharpened by cutting it obliquely to facilitate penetration of the duct directly, without previous puncture which might cause the duct to collapse.

(2) The head was attached to transluscent vinyl tubing (Portex Ltd) which was very soft and absorbed any shock to the external part of the cannula which might be conducted to the head, dislodging it from the duct (Fig. 1).

The animals were given 0·1 mg. Vit. K. before cannulation and fed 5% glucose in normal saline when lymphocytes alone were required, or were on water for experiments in which we used whole lymph or cell-free lymph fluid. The lymph

FIG. 1.—A two piece cannula suitable for thoracic duct cannulation of the rat.

was collected into 100 ml. siliconized sterile glass flasks, into which 1 ml. of heparin solution (250 units/ml.) was placed, the flask being agitated every few minutes.

*Immunotherapy procedures*

Lymph was collected from previously immunized rats every 12 hours from 3–5 days after boosting. The cell content was counted and cell viability was determined by the dye exclusion technique. Where washed lymphocytes were required these were obtained by centrifuging gently for 5 minutes; the supernatant was discarded and the cells were then washed in TC199 and resuspended in the same medium to contain the required number of lymphocytes per therapy dose (in not more than 1 ml. of medium). Although these rat lymphocytes seemed perfectly viable, we were unsuccessful in attempts to grow them *in vitro* in tissue culture, and thus were never able to test them for lymphocyte transformation against the mouse cells.

In preliminary experiments, fresh washed lymphocytes were given either intravenously or intraperitoneally in one dose (either 25, 50 or 100 $\times$ $10^6$) into mice which had been previously transplanted with 100–250,000 tumour cells. Treatment given at intervals from 0–5 days after transplantation was tried, without success. When it was shown that whole rat lymph could be effective the following experiments were made, initially with a short period of *in vitro* mixing

(to ensure complete contact of all components) before inoculation, and finally with *in vivo* therapy of the tumour.

### *The* In Vitro *Effect of Rat Thoracic Duct Lymph*

Fresh tumour cells were always used; these were gently centrifuged, the supernatant was discarded and the cells were resuspended in saline to contain $5 \times 10^6$ cells per ml.

Incubation was done in sterile siliconized glass universals at room temperature. The lymph used was always fresh from within 4–6 hours of collection.

*Experiment* 1—*The effect of whole lymph fluid*

In this experiment $10^6$ tumour cells in 0·2 ml. saline were incubated for 10 hours with 0·8 ml. whole lymph which contained $40 \times 10^6$ lymphocytes per ml. freshly collected from an immunized rat. Similarly tumour cells were incubated with whole lymph from an unimmunized rat. At the same time the same number of tumour cells were incubated with 0·8 ml. normal saline in a similar bottle as control. Then 0·1 ml. of the mixture from each bottle, containing $10^5$ tumour cells, was transplanted into each of groups of 5 mice.

*Experiment* 2—*The effect of washed lymphocytes or cell-free lymph*

In this experiment $10^6$ tumour cells in 0·2 ml. saline were transferred into each of 4 bottles (A, B, C, D) to which the following additions were made: (A) 0·8 ml. saline; (B) 0·8 ml. of whole lymph fluid containing $250 \times 10^6$ lymphocytes from an immunized rat; (C) 0·8 ml. of lymphocyte-free lymph from an immunized rat; (D) $250 \times 10^6$ lymphocytes from an immunized rat: they were washed and re-suspended in 0·8 ml. normal saline. After 10 hours incubation, 0·1 ml. from each bottle was transplanted into each of groups of 5 mice.

*Experiment* 3—*Determination of the effective fraction after separation through Sephadex G*200

Twelve ml. of cell-free lymph from an immunized rat was applied to a Sephadex G200 column. The three main peaks were collected and concentrated by vacuum ultrafiltration down to the original volume of the lymph. The fractions were then dialysed against phosphate buffered saline for 48 hours at 4° C. and finally sterilized by millipore filtration. Into 5 bottles (E, F, G, H, I) $10^6$ tumour cells in 0·2 ml. saline were transferred and the following additions were made: (E) 0·8 ml. normal saline; (F) 0·8 ml. fraction 19s; (G1) 0·8 ml. fraction 7s; (H) 0·8 ml. fraction 4s; (I) 0·8 ml. whole lymph.

Subsequently bottle G2 was set up using 0·8 ml. of fraction 7s which had been recycled through Sephadex G200 to free it from the originally contaminating tail of the 19s fraction.

Each bottle was then incubated for 10 hours at room temperature, and thereafter 0·1 ml. ($10^5$ tumour cells) was inoculated, using groups of 5 mice for each bottle.

### *The* In Vivo *Effect of* 19*s Fraction from Immune Rat Lymph*

*Experiment* 4

Four groups (J, K, L, M) of 5 mice were inoculated with $10^5$ tumour cells. (J) was left as the control; (K) received 0·5 ml. of 19s fraction intraperitoneally 30

minutes after inoculation; (L) received 0·5 ml. doses of 19s fraction at 30 minutes, 1 day, 2 days and 3 days after inoculation; (M) was given 0·5 ml. of 19s fraction at 6 days, 7 days and 8 days after inoculation:

*Controls*

Throughout the above experiments the following controls were used:

(i) Tumour cells in saline without any other addition were inoculated to check the viability and take of the tumour being used.

(ii) Whole thoracic duct lymph or washed lymphocytes from unimmunized litter mates of the immunized rats were used to screen for any natural immune reaction.

(iii) Mice not inoculated with tumour were given the lymph (or its products) from immunized rats to check for any obvious side-reactions against the normal tissues of the mouse.

*Mode of action of 19s fraction*

The *in vitro* studies (exp. 3, F) were repeated, also using 19s fraction heated to 56° C. for half an hour to see if complement (also contained in the front peak from Sephadex G200) was necessary.

Trypan blue exclusion studies were made of both MP 5563 cells and normal C3H mouse cells (from peritoneal washings or from dextranized blood) before and after incubation with active 19s fraction.

### RESULTS

Using the survival as a criterion, the preliminary *in vivo* therapy using washed lymphocytes (from immunized rats) in different doses and at different time intervals after transplantation did not cause any marked difference whether they were given intravenously or intraperitoneally. Dye exclusion studies had shown that rat lymphocytes were still 95% viable after washing.

TABLE I.—*Results of Experiment* 1; In Vitro *Effect of Immune Rat Thoracic Duct Lymph Against MP* 5563

| | | Thoracic duct lymph from | |
|---|---|---|---|
| Incubation with | Saline control | Normal rat | Immunized rat |
| Survival | All dead within 14 days | All dead within 14 days | All alive at 6 months |

TABLE II.—*Results of Experiment* 2; *the* In Vitro *Effect of Immune Cell-free Lymph Against MP* 5563

| | | Immune rat lymph | | |
|---|---|---|---|---|
| Incubation with | Saline control | Whole lymph | Cell free lymph | Washed viable lymphocytes 250 per tumour cell |
| | A | B | C | D |
| Survival | All dead within 14 days | All alive at 5 months | All alive at 5 months | All dead within 14 days |

TABLE III.—*Results of Experiment 3; the Fraction of Cell-free Lymph Effective* In Vitro *Against MP* 5563

| | | Sephadex G200 fraction of immune rat lymph | | | | |
|---|---|---|---|---|---|---|
| Incubated with | Saline control E | 19s F | First 7s (+ trail 19s) G1 | Recycled 7s (no 19s) G2 | 4s H | Whole lymph I |
| Survival | All dead within 14 days | All alive at 4 months | 3/5 mice developed the tumour and died within 30 days. 2/5 alive at 4 months | All dead within 14 days | All dead within 14 days | All alive at 4 months |

TABLE IV.—*Results of Experiment 4; the* In Vivo *Effect of Immune* 19*s Fraction at Various Intervals After Inoculation of MP* 5563

| Control no treatment | 0·5 ml. at 30 min. only | 0·5 ml. at 30 min. and on days 1, 2 and 3 | 0·5 ml. on days 6, 7 and 8 |
|---|---|---|---|
| J | K | L | M |
| All dead within 14 days | All dead, average survival 26 days | All dead, average survival 26 days | All dead within 16 days |

The results of experiments 1–4 are shown in Table I–IV. Throughout, the controls revealed (i) that $10^5$ of the tumour MP 5563 cells were regularly lethal within 14 days (ii) that rat lymph from unimmunized litter mates was without effect and (iii) that in the doses used the immune rat lymph showed no obvious side effects on normal C3H mice.

From the above results it can be concluded that the effect of immune rat lymph is confined solely to the 19s cell-free fraction. With the guaranteed contact of prior *in vitro* incubation this 19s fraction can completely eliminate the successful take of MP 5563. *In vivo* its effect is as yet only to prolong survival, and even this effect seems largely confined to the first dose given 30 minutes after the inoculation of MP 5563. Doses thereafter have only a minimal effect.

The 19s fraction was ineffective after heat inactivation, so presumably requires complement for its action. The trypan blue exclusion studies revealed almost total killing of the MP 5563 cells after incubation *in vitro*, but also showed 85% killing of the normal C3H white cells from either peritoneal washings or peripheral blood.

### DISCUSSION

Lymphocytes from immunized rats had no effect on the mouse tumour (*in vivo* or *in vitro*), in spite of the large doses given (up to 500 lymphocytes per tumour cell) and although they had been collected at the time when the lymph was expected to contain a high proportion of immunoblasts (8–12%) (Delorme *et al.*, 1969).

The purpose of injecting the lymphocytes between day 0 and day 5 after transplantation was to attack the tumour before it became established and to show any synergism between the injected rat lymphocytes and any immune reaction of the mouse, such as that found by Alexander *et al.* (1967). The intravenous or intraperitoneal injection of the lymphocytes did not show any effect. The differences in the nature of the two experimental tumours might account for the differences in the effectiveness of the rat lymphocytes. The present tumour had originally arisen spontaneously in mice, whereas that of Alexander's team had been chemically induced.

The ineffectiveness of the washed rat lymphocytes to reject the tumour led us to look for any cofactor which might be necessary for the action of the lymphocytes. The cytotoxic effect found was shown later to be mediated only by the 19s fraction which presumably contained IgM antibodies. This reaction was inactivated by heating to 56° C. and presumably involved the fixation of complement. The partial cytotoxic effect of the first 7s fraction was shown to be due to the presence of some 19s fraction in it, tailing from the first peak. This effect was abolished when the 7s fraction was recycled through the column and freed from any contamination from the 19s fraction.

The possible application of the 19s fraction in therapy of the plasmacytoma was considered in experiment 4. The results would depend upon whether this antibody was directed specifically against the tumour or against normal tissue histocompatibility antigens as well. It does seem from the final trypan blue studies that the 19s fraction has both activities. It may not be all used up by the normal tissues of the mouse, although this does diminish its effectiveness. While as yet the mice are not obviously harmed by the doses given, preliminary results indicate the normal cells of the mouse are so vulnerable that larger doses of unadsorbed 19s fraction would be harmful. It also seems that the first dose is the only one that is really effective and that subsequent doses do not prolong survival further (see Table IV). This might suggest that the mouse forms an antibody against this rat 19s fraction which prevents its further effectiveness. The effect of the first dose on an established tumour *in vivo* was not of curative value, although the tumour could be killed *in vitro*. The *in vivo* dose may have been too small to be effective on a well grown tumour or alternatively the late tumour might have become protected *in vivo* by an enhancing antibody, etc. Adsorption studies will be undertaken to try and produce a tumour-specific antibody, so that a larger dose may be given and it is hoped to also encourage a cellular immune response from the rat by modifying the tumour antigen.

This investigation has been supported by a grant from the British Empire Cancer Campaign. We are most grateful to Dr. E. J. Delorme for his helpful instruction with the rat techniques, and to Dr. H. Valdimarsson's cooperation with lymphocyte studies.

### REFERENCES

Alexander, P., Connell, D. I. and Mikulska, Z. B.—(1966) *Cancer Res.*, **26**, 1508.

Alexander, P., Delorme, E. J., Hamilton, L. D. G. and Hall, J. G.—(1967) *Nature, Lond.*, **213**, 569.

Alexander, P. and Hamilton Fairley, G.—(1967) *Br. med. Bull.*, **23**, 86.

Delorme, E. J. and Alexander, P.—(1964) *Lancet*, ii, 117.

Delorme, E. J., Hodgett, J., Hall, J. G. and Alexander, P.—(1969) *Proc. R. Soc.*, B., **174**, 229.

Fakhri, O.—(1970) *Br. J. Cancer.*, **24**, 389.

Fakhri, O. and Hobbs, J. R.—(1970) *Br. J. Cancer.*, **24**, 395.

Gorer, P. A. and Amos, D. B.—(1956) *Cancer Res.*, **16**, 338.

HELLSTRÖM, K. E. AND MÖLLER, G.—(1965) *Prog. Allergy*, **9**, 158.
IRVIN, G. L., EUSTACE, J. C. AND FAHEY, J. L.—(1967) *J. Immun.*, **99**, 1085.
JANOWSKY, D. S., ROSENAU, W. AND MOON, H. D.—(1964) *Proc. Soc. exp. Biol. Med.*, **115**, 77.
KLEIN, E. AND KLEIN, G.—(1964) *J. natn. Cancer Inst.*, **32**, 547.
OLD, L. J. AND BOYSE, E. A.—(1964) *A. Rev. Med.*, **15**, 167.
SOUTHAM, C. M. AND DIZON, Q. S.—(1969) *Cancer Res.*, **29**, 1428.